# Multi-Scale Feature Integrated Attention-Based Rotation Network for Object Detection in VHR Aerial Images

**DOI:** 10.3390/s20061686

**Published:** 2020-03-18

**Authors:** Feng Yang, Wentong Li, Haiwei Hu, Wanyi Li, Peng Wang

**Affiliations:** 1 Key Laboratory of Information Fusion Technology, Ministry of Education, Northwestern Polytechnical University, Xi’an 710100, China; liwentong@mail.nwpu.edu.cn (W.L.); huhaiwei@mail.nwpu.edu.cn (H.H.); 2 Institute of Automation, Chinese Academy of Sciences, Beijing 100190, China; peng_wang@ia.ac.cn

**Keywords:** object detection, aerial images, feature attention, convolutional neural networks (CNNs)

## Abstract

Accurate and robust detection of multi-class objects in very high resolution (VHR) aerial images has been playing a significant role in many real-world applications. The traditional detection methods have made remarkable progresses with horizontal bounding boxes (HBBs) due to CNNs. However, HBB detection methods still exhibit limitations including the missed detection and the redundant detection regions, especially for densely-distributed and strip-like objects. Besides, large scale variations and diverse background also bring in many challenges. Aiming to address these problems, an effective region-based object detection framework named Multi-scale Feature Integration Attention Rotation Network (MFIAR-Net) is proposed for aerial images with oriented bounding boxes (OBBs), which promotes the integration of the inherent multi-scale pyramid features to generate a discriminative feature map. Meanwhile, the double-path feature attention network supervised by the mask information of ground truth is introduced to guide the network to focus on object regions and suppress the irrelevant noise. To boost the rotation regression and classification performance, we present a robust Rotation Detection Network, which can generate efficient OBB representation. Extensive experiments and comprehensive evaluations on two publicly available datasets demonstrate the effectiveness of the proposed framework.

## 1. Introduction

An insightful understanding in very high resolution (VHR) aerial images (20 cm–30 cm resolution) can be available under imagery analysis for geospatial areas [1,2]. Object detection, a critical component of automatic aerial imagery analysis, plays an important role in national defense construction, urban planning, environmental monitoring and so on [3,4,5]. Although many object detection methods in VHR aerial images have been proposed before, this task is still full of great challenges due to arbitrary orientations and large scale variations with various background. Recently, with the rapid development of convolutional neural networks (CNNs), a variety of CNN-based object detection frameworks [6,7] have been proposed and very impressive results have been achieved over natural benchmarks including PASCAL VOC [8] and MS COCO [9]. The existing CNN-based object detection methods can be commonly divided into two parts: two-stage methods and single-stage methods. In the two-stage methods, the input image in the first stage contributes to the generation of category-independent region proposals, and subsequently features of these regions are extracted, then the refinement of category-specific classifiers and regressors is achieved for classification and regression in the second stage. Finally, accurate detection results are attained with the deletion of redundant bounding boxes, such as non-maximum suppression (NMS). Region-based CNN (R-CNN) [10] is a pioneering work. Its reformative version SPP-Net [11], Fast-RCNN [12] makes it possible to simplify learning and runtime efficiency. Faster-RCNN [6] integrates the proposed Region Proposal Network (RPN) and Fast R-CNN into a unified network by sharing convolution weights, which makes object detection quick and accurate in an end-to-end manner. Zhang et al. [13] proposed a multiscale cascaded object detection network and introduce multiscale features in pyramids to obtain feature of each scale with a novel attention method, which can highlight the object features and efficiently detect objects for traffic sign with complex background. Many high-performance detection methods are also proposed until now, such as FPN [14], R-FCN [15], Mask-RCNN [16], Libra RCNN [17], Trident-Net [18], and so forth. In addition, the single-stage methods directly consider object detection as a regression problem, without the procedure of proposal generation, which can perform nearly real-time achievement. YOLO [19] and SSD [20] are popular in single-stage methods, which maintained real-time speed with ensured detection accuracy. RetinaNet [7] proposes a new focal loss function to address class imbalance issue of single-stage approaches. Inspired by the two-stage methods, RefineDet [21] can adjust the sizes of anchors and locations with the adoption of cascade regression and the application of an Anchor Refinement Module (ARM), and then filter out easy negative anchors to improve accuracy.

Inspired by the great success of CNN-based object detection methods in natural images, a growing number of studies have been devoted recently to object detection in VHR optical aerial images [22,23]. Considering the arbitrary orientation, Cheng et al. [24] proposed to learn a Rotation-Invariant CNN (RICNN) model based on R-CNN framework used for multi-class object detection. To achieve real-time object detection, Tang et al. [25] adopted a regression method based on SSD to detect vehicle targets through applying a set of default boxes with various scales on per feature map location. To be specific, to better fit the shape of objects, the offsets for each default box are predicted. To deal with the problem of multi-scale detection with the large ratio of remote sensing objects, Guo et al. [26] proposed a unified multi-scale framework, which is composed of multi-scale object proposal network and a multi-scale detection network. To achieve further accuracy of the localization in aerial images, Zhang et al. [27] proposed a Double Multi-scale Feature Pyramid Network (DM-FPN), which makes the most of semantic and resolution features simultaneously and bring up some multi-scale training, inference and adaptive categorical non-maximum suppression (ACNMS) strategies. In addition, object detection based on weakly supervised deep learning method arouses more and more attentions of researchers in recent years. Except depending on the costly bounding box annotations, Li et al. [28] proposed a weakly supervised deep learning method which combine the separate category information and mutual cues between scene-level pairs to train a deep network for multi-class geospatial object detection.

**Figure 1:**
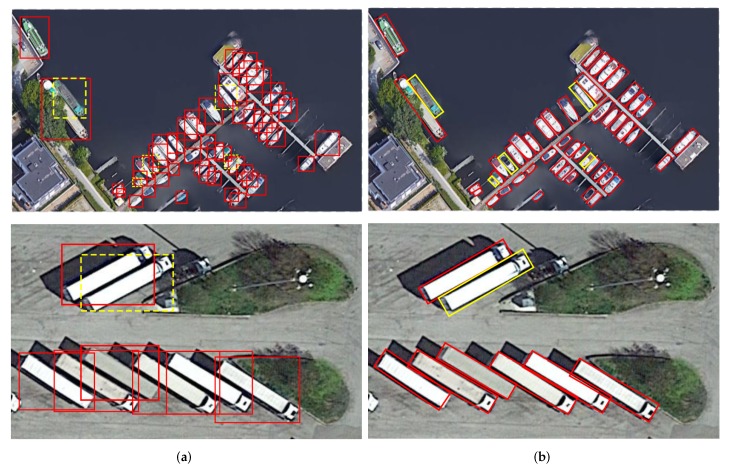
(**a**) HBB detection of densely-distributed and strip-like objects with large redundancy regions; objects in yellow line of dashes are prone to missed detection; (**b**) OBB detection is suitable; objects in yellow line can be detected rightly with fitting regions.

These methods have achieved very promising detection performances by using horizontal bounding boxes (HBBs) as region of interests. HBBs are appropriate for ground-level images with mainly regular and vertical objects. However, in VHR aerial images, objects can have any orientations between 0 and 360 degrees viewed from overhead. Such HBB-based methods can result in missed detection and redundancy of detection region especially for densely-distributed and strip-like objects such as ship and large vehicle as shown in Figure 1. Therefore, employing oriented bounding boxes (OBBs) as region of interests is highly recommended, which can identify more accurate and intuitive localization with fitting regions for aerial images. Recently, OBB-based methods in VHR aerial images gradually attract researchers’ attention. The existing methods, which contribute to the oriented object detection, can be divided into three categories: OBB detection with generating rotated region proposals, OBB regression from the coarse horizontal region proposals and OBB representation by calculating minimum area rectangle from the mask shape prediction. For the first method, Yang et al. [29] presented a Rotation Dense Feature Pyramid Networks (R-DFPN) by producing rotational proposals from the RPN to achieve rotated location regression in the Fast-RCNN stage for ship detection with a large aspect ratio. Azimi et al. [30] proposed Rotation Region Proposal Network (R-RPN) and Rotated Region of Interest Network (R-RoI) to generate and handle Rotation-based proposals respectively. Ding et al. [31] proposed RoI Transformer to address the misalignment problem between the horizontal region of interests and oriented objects. Moreover, the Rotated RoI Learner and Rotated Position Sensitive RoI Align layer are designed to boost rotated object classification and regression. These methods achieve advanced performance. Meanwhile, the computational burden will mount as well as a result of the possibility that each pixel may generate dozens or even hundreds of rotated proposals with using a more complicated structure. For the second method, R${}^{2}$CNN [32] is an efficient and classic rotation detection method, but it is especially for scene text detection, which is not suitable for aerial scenarios. Inspired by the R${}^{2}$CNN, Yang et al. [33] proposed a multi-class detection method based on Faster R-CNN named SCRDet, making it probable to estimate and regress OBBs by making good use of the coarse resolution information in horizontal regions. SCRDet can achieve precisely rotation detection especially for small and cluttered objects on VHR remote sensing images. For the third method, Li et al. [34] proposed a rotation detector named RADet inspired by Mask RCNN [16], which obtains the rotated bounding box by calculating the minimum aera rectangle from the correspondingly precited mask shape. This simple presentation with an efficient multi-scale network achieve the competent performance on two benchmarks DOTA [35] and NWPU VHR-10 [36].

To better build an accurate and oriented object detection for multi-class objects in VHR aerial images with diverse background, this paper proposes a novel Multi-scale Feature Integration Attention Rotation Network (MFIAR-Net). The proposed framework is composed of three modules: Multi-scale Feature Integration Network (MFIN), Double-Path Feature Attention Network (DPFAN) and Rotation Detection Network. Compared with advanced rotation detection methods such as RADet [34], SCRDet [33], RoI-Transformer [31] and ICN [30], our framework is more suitable for multi-class and arbitrary-oriented object detection in aerial images. The main contributions of this paper are as follows:

- We propose an accurate and unified Multi-scale Feature Integration Attention Rotation Network (MFIAR-Net) for VHR aerial images, which can efficiently detect the multi-category and arbitrary-oriented objects with fitting OBBs.
- We propose a Multi-scale Feature Integration Network (MFIN) by integrating semantically strong, low-spatial resolution features and semantically weak, high-spatial resolution features into a discriminative feature map to handle the scale variations of geospatial objects. The Asymmetric Convolution Block (AC Block) is a crucial design to substitute for standard square-kernel convolutional layer to extract distinguished features.
- We design a Double-Path Feature Attention Network (DPFAN) supervised by the mask information of ground truth to guide the network to focus on object representations and suppress the irrelevant background information.
- We present a robust Rotation Detection Network to regress OBBs with five parameters (x,y,w,h,θ), in which Position Sensitive RoI Align (PS RoI Align) layer and a new multi-task learning loss is introduced to make the localization of deep network more sensitive and benefit the oriented regression.

Besides, the multi-scale training and inference strategy is adopted to handle multi-scale remote sensing objects. The proposed framework is evaluated on two publicly aerial datasets DOTA [35] and HRSC2016 [37] compared with several state-of-the-art approaches. And the effectiveness and superiority of the proposed framework is demonstrated with comprehensive experiments.

The rest of this paper is organized as follows: Section 2 describes the proposed MFIAR-Net for oriented object detection in detail. Section 3 illustrates the datasets, details of implementation, evaluation criteria and experiment results. Section 4 discusses the proposed framework, conducts the careful ablation study and analyzes limitations and future research directions. Finally, the conclusions are drawn in Section 5.

## 2. Proposed Method

**Figure 2:**
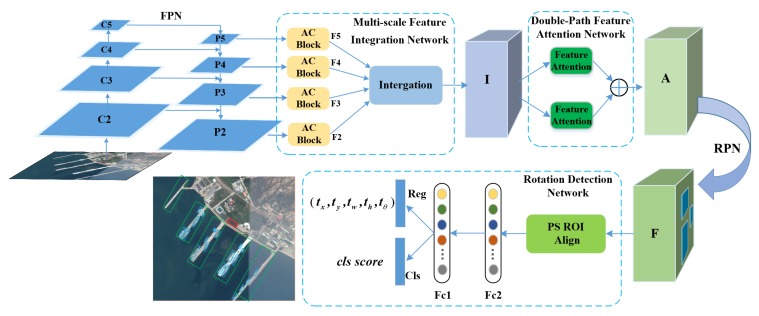
Overview framework of Multi-scale Feature Integration Attention Rotation Network (MFIAR-Net) for oriented object detection. The proposed framework based on FPN, consists of Multi-scale Feature Integration Network, Double-Path Feature Attention Network and Rotation Detection Network.

In this section, we present details of the proposed Multi-scale Feature Integration Attention Rotation Network (MFIAR-Net). Figure 2 shows the overview framework of MFIAR-Net. First of all, the feature map is expected to contain more multi-scale feature information by FPN and Multi-scale Feature Integration Network (MFIN). The Double-Path Feature Attention Network (DPFAN) can guide the network to focus foreground information. The coarse horizontal regions are still regressed in the end of first stage to keep critical information. To improve the sensitivity of location and the precision of five-parameter (x,y,w,h,θ) regression in Rotation Detection Network, PS RoI Align layer and a new Multi-task learning loss is introduced. More details are provided in the following subsections.

### 2.1. Multi-Scale Feature Integration Network (MFIN)

Feature Pyramid Network (FPN) [14] is an effective method to extract multi-scale feature information from a single image. In order to obtain distinguished feature representation of FPN for geospatial objects, Asymmetric Convolution Block (AC Block) [38] is employed after the output of each scale. Furthermore, we integrate simultaneously the multi-scale feature maps into a discriminative feature map with appropriate size, the integrated feature possesses balanced information from each spatial resolution, which is key for scale variations in aerial images. MFIN consists of two important processes: multi-scale feature extraction and multi-scale feature integration.

#### 2.1.1. Multi-Scale Feature Extraction

**Figure 3:**
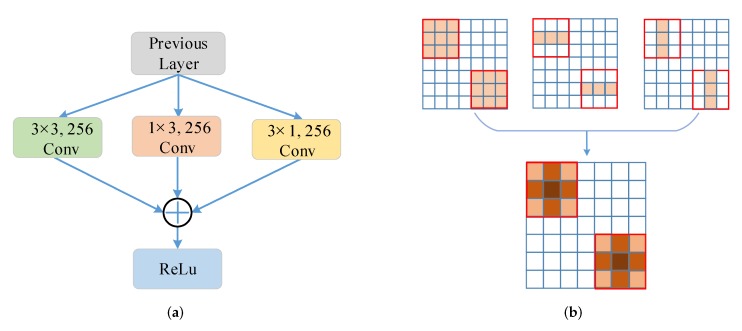
(**a**) The structure of Asymmetric Convolution Block (AC Block) with three parallel convolution layers with 3×3, 3×1 and 1×3 kernels. The ReLu activation is operated on the sum of them.; (**b**) The AC Block focuses on the significance of skeletons feature of ‘+’ shape.

**Figure 4:**
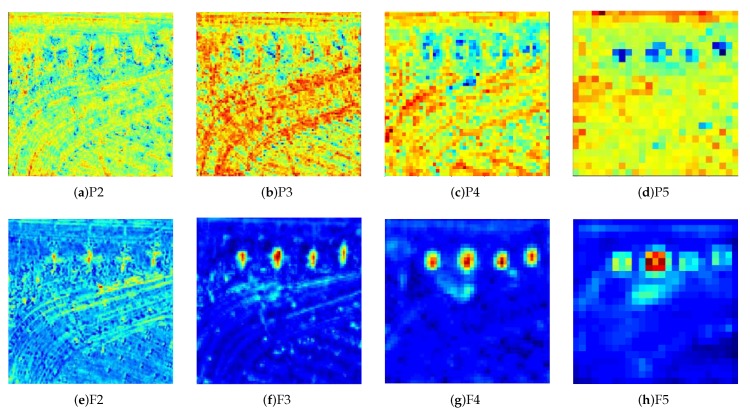
(**a**)–(**d**) represent the Feature map P2, P3, P4 and P5 respectively, (**e**)–(**h**) represent the corresponding F2, F3, F4 and F5 after AC Block.

As Figure 2 shows, the proposed architecture can be divided into five stages based on ResNet [39], and the output of each stage’s final residual block is regarded as C2, C3, C4, C5 with diverse spatial resolutions. Note that the strides are 4, 8, 16, 32 pixels corresponding to the input image. The P2, P3, P4, P5 are obtained by a top-down pathway and lateral connections corresponding to C2, C3, C4, C5 respectively. Concretely, the top-down pathway begins with the bottom layer of the network and progressively upsamples it while transformed versions of higher-resolution features are added from the bottom-up pathway. FPN generates semantically strong, low-spatial resolution features and semantically indistinctive, high-spatial resolution features in different level from P5 to P2. To remove the mixture problem of upsampling and obtain distinguished features of P2, P3, P4, P5, the AC Block with three parallel branches is introduced to replace the standard square-kernel 3×3 convolution layer. As Figure 3 shows, the AC Block is constructed of three parallel convolutional layers with 3×3, 1×3 and 3×1 kernels respectively. Different from the Inception Module [40], the outputs of them are summed up to enrich the feature representation’s space, then a ReLu activate function is adopted to get the output feature. In AC Block, the horizontal 1×3 kernel and vertical 1×3 kernel are added, which concentrates on the significance of skeleton feature of ‘+’ shape, especially the features of object center. Because the skeleton of ‘+’ shape have key information of object and the center point has the maximum energy. As Figure 4 illustrates, the output of FPN features including P2, P3, P4, P5 have the blurred objects. The objects of interests are highly distinguished in F2, F3, F4, F5 after the AC Block. Compared with the standard 3×3 convolution, AC Block delivers more powerful feature for the multi-scale feature pyramid with little extra time-consume computations. The ablation study about AC Block also demonstrates its effects.

#### 2.1.2. Multi-Scale Feature Integration

**Figure 5:**
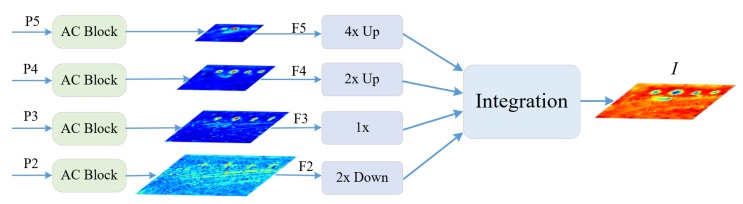
The structure of MFIN. Based on FPN, the AC Block is adopted to replace 3×3 convolution layer. To make the integration of multi-scale features, different resized operations are employed. Note that Up represents upsample layer and Down represents downsample layer, which are implemented by bilinear interpolation method.

The FPN plays an important role in multi-scale feature extraction. Inspired by the “feature balance” from the Libra RCNN [17]. The FPN method will make fused features focus more on adjacent resolution but less on others. The semantic information contained in non-adjacent levels would be diluted once per fusion during the information flow. So, our study focuses on the good manner of feature balance designed for remote sensing images. The multi-level features F2, F3, F4, F5 are strongly attained after the AC Block. Low-level and high-level informations are complementary for object detection. How to make full use of multi-level features to generate more discriminative representations is crucial to detection performance. As indicated in Figure 5, F2 possesses the highest spatial resolution, F5 possesses the lowest spatial resolution. Aiming to integrate multi-level features and maintain their semantic hierarchy concurrently, we first resize the multi-level features F2, F3, F4, F5 to an intermediate size by using bilinear feature interpolation method. By introducing the expected size of integrated features, SCRDet [33] explored that the small stride of the anchor ($S_{A}$) can obtain more samples with high quality particularly for the small objects. Meanwhile, $S_{A}$ is equivalent to the reduction factor of the feature map relative to the input image. In order to balance the semantic information and location information, we adopt the size of F3 as the integrated size. The integration feature can be obtained by the averaging operation as Equation (1). In this procedure, multi-scale features from high-level to low-level are aggregated at the same time. The output of MFIN integrated semantic and spatial features to strengthen the multi-scale information flow and make features more balanced, which is denoted as *I*.

(1) $$I=\frac{1}{4}\sum_{F_{2}}^{F_{5}}F_{i}$$

### 2.2. Double-Path Feature Attention Network (DPFAN)

**Figure 6:**
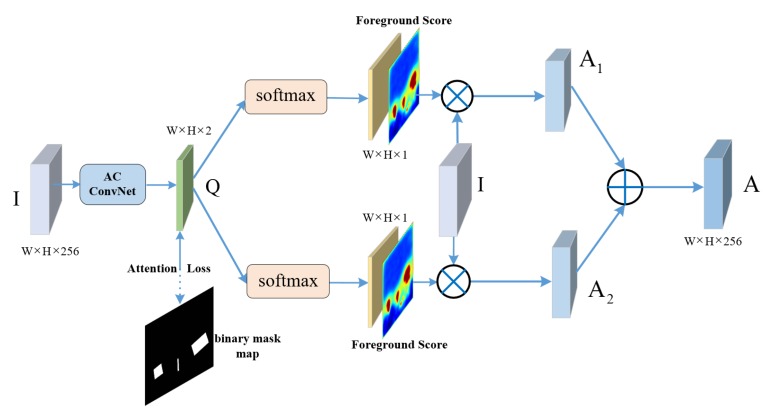
The structure of DPFAN, a double-path feature attention network supervised by binary mask map of ground truth in training time. In inference time, the model works without mask map.

Aerial images have varied background of geospatial scenarios. Excessive background noise causes the object information inconspicuous, which results in missed detection and false alarms. Visual attention turns out to be effective in various computer vision tasks [13,41,42]. We construct a supervised Double-Path Feature Attention Network (DPFAN) to guide the whole network to capture the object information in visual representation. Figure 6 shows the detailed structure of DPFAN. Concretely, the feature map I passes through an AC ConvNet structure, and a new two-channel feature map Q is generated. In AC ConvNet, the AC Block still performs better than standard 3×3 convolution in obtaining strong representation and improving robustness of rotation work. Then we take two parallel branches to exact the foreground features. In each branch, the softmax function is employed on Q to obtain the saliency map, which represents the scores between [0,1] of foreground and background in different channels. We only select the foreground score map to multiply with I to attain the foreground regions, which are $A_{1}$ and $A_{2}$ respectively. Finally, a better attention feature map *A* is obtained by summing up $A_{1}$ and $A_{2}$ from the two parallel attention branches. The implementation process of feature attention is represented as Equation (2). Note that the index 0 denotes the foreground score map.

(2) $$A=A_{1}+A_{2}={\left[soft\max_{1}\left(Q\right)\right]}_{0}*I+{\left[soft\max_{2}\left(Q\right)\right]}_{0}*I$$

To guide the network with a definite direction, we adopt a supervised learning method. According to ground truth, a binary mask map can be obtained as a label, and then the attention loss between the binary mask and two-channel feature map Q can be calculated by using the pixel-wise cross-entropy loss only in training time. In inference time, our model can work effectively and independently without mask information of ground truth.

### 2.3. Rotation Detection Network

**Figure 7:**
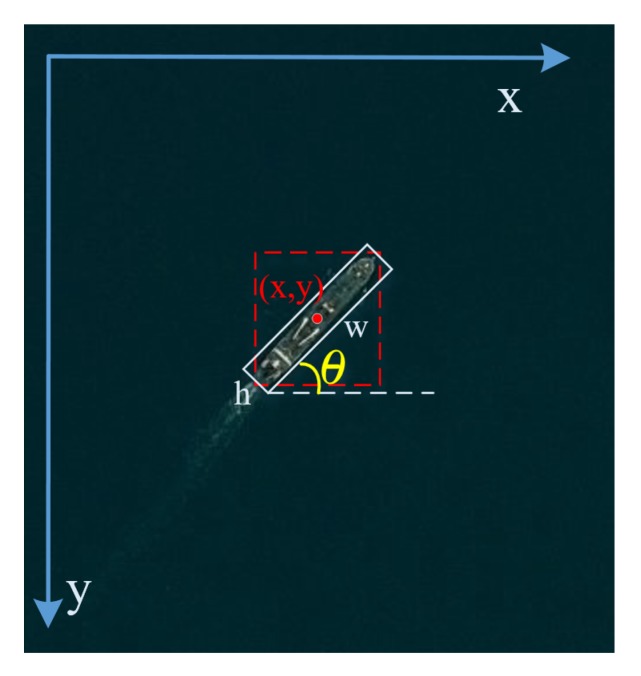
The illustration for the rotation angle. Red line of dashes represents the HBB; White line represents the OBB. θ represents the rotated angle between the x-axis and w side.

Based on Faster R-CNN [6], the proposed network has two stages. In first stage, the coarse horizontal region proposals are still obtained from RPN. In the second stage, the final predicted OBBs with five parameters (x,y,w,h,θ) from the rotation detection head are regressed. Specially, as Figure 7 shows, θ is defined as the angle between the x-axis and w side in range [−π/2,0). (x,y) means the oriented box’s center coordinate and *w*, *h* represent the width and height of oriented box, respectively. Besides, rotation non-maximum suppression (R-NMS), the rotational variant of NMS, is applied to select the best localized regions and to remove redundant detections for OBBs. In order to build a robust rotation detection with a cute angle θ, PS RoI Align layer and a new multi-task learning loss function are introduced in the proposed Rotation Detection Network.

#### 2.3.1. PS RoI Align Layer

A fixed-length (e.g., 7×7) feature map for succeeding classification and bounding box regression tasks is built by RoI pooling layer [12] in the second stage. Considering the problem of feature misalignment especially for large aspect ratio objects, R-DFPN [29] and SCRDet [33] employed RoI Align [16] by utilizing bilinear interpolation to obtain the precise coordinates instead of quantified integers for candidate regions. To settle a dilemma between translation-invariance in image classification and translation-variance in object detection, PS RoI Pooling is proposed in R-FCN [15]. At first, a fully convolutional network generates position-sensitive score maps. Each of these score maps encodes the position information in reference to a relative spatial position. Then, a positive-sensitive RoI pooling layer is followed which gets score maps information in the final convolutional layer of second stage.

In the proposed method, a more robust PS RoI Align layer is built by combining the RoI Align with PS RoI Pooling. Specially, we abandon two rounding operations during the PS RoI Pooling, one is to quantify the coordinates of candidate region into integer at first, the other is to quantity the coordinates of each bins in position-sensitive score maps. Like RoI Align, we use bilinear interpolation to obtain the precise coordinates respectively. Not only can PS RoI Align avoid the misalignment between the inputs and the extracted feature maps, but also can make the deep convolutional backbone effectively converted to object detector with well translation-variance performance. The experiments demonstrate that PS RoI Align layer can improve the performance 1.27% and 0.72% than frequently used RoI Pooling layer and RoI Align layer respectively.

#### 2.3.2. Multi-Task Learning Loss Function

In our proposed MFIAR-Net, the tasks including coarse horizontal region proposals learning in RPN, regression and classification of OBBs and attention network learning are performed at the same time. We present a new multi-task learning loss to guide the whole framework as the objective function, which is defined as:

(3) $$L=L_{RPN}+\lambda_{1}L_{Reg\_OBB}+\lambda_{2}L_{Cls}+\lambda_{3}L_{Att},$$

where $L_{RPN}$ indicates the region proposal networks loss, $L_{Reg\_OBB}$ represents the oriented bounding box loss, $L_{Cls}$ means the classification loss and $L_{Att}$ is the loss of attention network learning in DPFAN. $\lambda_{1}$, $\lambda_{2}$ and $\lambda_{3}$ balance losses between the different tasks.

Specially, $L_{Reg\_OBB}$ is defined as:

(4) $$L_{Reg\_OBB}=\frac{1}{N}\sum_{i}p_{i}^{*}\sum_{j\in \{x,y,w,h,\theta \}}L_{reg}\left(v_{ij},{v_{ij}}^{*}\right),$$

when the labeled class is foreground, $p_{i}^{*}=1$, else $p_{i}^{*}=0$. $v^{*}=\left(v_{x}^{*},v_{y}^{*},v_{w}^{*},v_{h}^{*},v_{\theta }^{*}\right)$ denotes the target vector of ground truth, $v=(v_{x},v_{y},v_{w},v_{h},v_{\theta })$ indicates the predicted offset vector. $L_{reg}$ is smooth L1 function, which is responsible for OBB’s regression of five parameters. They are defined respectively as follows:

(5) $$\begin{array}{cc} L_{reg} & =smooth_{L_{1}}\left(v-v^{*}\right)\\ smooth_{L_{1}}\left(x\right) & =\left\{\begin{array}{c} 0.5x^{2},if\left|x\right|<1\\ \left|x\right|-0.5,otherwise \end{array}\right. \end{array}$$

(6) $$\begin{array}{cc} v_{x} & =\left(x-x_{a}\right)/w_{a},v_{y}=\left(y-y_{a}\right)/h_{a}\\ v_{w} & =\log\left(w/w_{a}\right),v_{h}=\log\left(h/h_{a}\right)\\ v_{\theta } & =\theta -\theta_{a} \end{array}$$

(7) $$\begin{array}{cc} v_{x}^{*} & =\left(x^{*}-x_{a}\right)/w_{a},v_{y}^{*}=\left(y^{*}-y_{a}\right)/h_{a}\\ v_{w}^{*} & =\log\left(w^{*}/w_{a}\right),v_{h}^{*}=\log\left(h^{*}/h_{a}\right)\\ v_{\theta }^{*} & =\theta^{*}-\theta_{a}, \end{array}$$

where x,y,w,h and θ indicate the box’s center coordinates, width, height and rotated angle, respectively. Variable *x*, $x_{a}$ and $x_{a}^{*}$ mean predicted box, anchor box and ground truth box respectively (similarly for *y*, *w*, *h*, θ). Besides, $L_{Att}$ in DPFAN guides the network to have a learning direction, which is defined as:

(8) $$L_{Att}=\frac{1}{h\times w}\sum_{i}^{h}\sum_{j}^{w}L_{att}\left(u_{ij},u_{ij}^{*}\right),$$

where $u_{ij}^{*}$ represents mask’s pixel of label, $u_{ij}$ represents predicted pixel of the two-channel feature map Q in the DPFAN. $L_{att}$ is pixel-level softmax cross-entropy loss. Note that $L_{RPN}$ and $L_{Cls}$ are the same as Faster R-CNN [6].

## 3. Experiments

In this section, we will demonstrate the effectiveness of the proposed MFIAR-Net on publicly available aerial datasets: DOTA [35] and HRSC2016 [37]. At fisrst, we introduce datasets, implementation details and evaluation metrics. Then we compare MFIAR-Net method with the state-of-art methods in accuracy and speed performance.

### 3.1. Dataset Description

#### 3.1.1. DOTA

In VHR optical aerial images with arbitrary quadrilateral annotation, the DOTA [35] is a large-scale dataset for benchmarking object detection captured from different sensors and platforms. DOTA involves 2806 aerial images totally with pre-divided 1411 training images, 458 validation images and 937 testing images. The fully annotated DOTA benchmark possesses 188,282 instances, which belong to 15 common classes, namely, plane (PL), baseball diamond (BD), bridge (BR), ground track field (GTF), small vehicle (SV), large vehicle (LV), ship (SH), tennis court (TC), basketball court (BC), storage tank (ST), soccer ball field (SBF), roundabout(RA), harbor(HA), swimming pool (SP) and helicopter(HC). The image size, ranging from around 800×800 to 4000×4000 pixels, contains objects which exhibit a great variety of scales, orientations, and shapes. The testing images have no annotations. The accuracy evaluation of the test data, accordingly, have to be submitted to the DOTA Evaluation Server http://captain.whu.edu.cn/DOTAweb/evaluation.html with a fixed format.

#### 3.1.2. HRSC2016

HRSC2016 [37] is a challenging dataset collected from famous harbors in Google Earth for ship detection. The dataset involves 1061 images totally with 436 images for training, 181 images for validation and 444 images for test respectively. The fully annotated HRSC2016 possesses 2976 samples with more than 20 categories of ships in diverse appearances. The image sizes range from 300×300 to 1500×900, most of which are larger than 1000×600. The strip-like ship detection is a better illustration of OBBs.

### 3.2. Implementation Details

Our designed MFIAR-Net is an end-to-end learning network by using ResNet-101 [39] as backbone, which is pretrained on ImageNet [43] to initialize the network. Our experiments are implemented by TensorFlow (https://tensorflow.org) on a Nvidia GeForce RTX 2080Ti GPU with 11G memory. For DOTA, we partition original images into 800×800 patches with 200 pixels’ overlap by a sliding window. The model is trained by 300k steps in total, and the learning rate is 3e-4 for the first 100k steps, then 3e-5 and 3e-6 for the next two 100k steps. For HRSC2016, we resize the short side of image to 800, the long side is fitted with the original ratio for training and testing time. We train the model with the same initial learning rate for the total 200k steps, the learning rate is 3e-4 for the first 80k steps, then 3e-5 and 3e-6 for the next 70k and 50k steps. The common settings for two datasets are as follow. We adopted the Momentum Optimizer as model optimizer where weight decay is set to 0.0001 and momentum is set to 0.9. The batch size of input image is set to 1, and the mini-batch size of RoIs in two-stage is 512 for training. For anchor details, the base anchor size is set to 256, and the anchors scales are setting from 2e-4 to 2e0. Besides, anchor ratios are set to [1/1, 1/2, 1/3, 1/4, 1/5, 1/6, 1/7, 1/9] to cover all objects’ size as far as possible. When IoU > 0.7, the anchor is regarded as a positive sample, and a negative sample is generated when IoU < 0.3. Besides, the two thresholds in the second stage get set to 0.4 owing to the sensitivity of angle and IoU with ortated rectangle. The balanced hyperparameters in Equation (3) are set to $\lambda_{1}=4$, $\lambda_{2}=2$, $\lambda_{3}=1$. It is worth noting that the setting of balanced hyperparameters is important for fully training of deep model. To a certain degree, it depends on empirical data in most cases. The initial values are set to 1 for $\lambda_{1}$, $\lambda_{2}$ and $\lambda_{3}$, respectively. The loss values including regression, classification and attention are vital reference data in training time. In this paper, the regression of five parameters (x,y,w,h,θ) is a primary task, and increasing $\lambda_{1}$ can enhance performance of OBB regression. The classification is also important for OBB localization. Increasing $\lambda_{2}$ in a small degree can also strength the performance of model. Attention is mainly aimed for feature extraction, which can make indirect effects for OBB regression. The balanced parameter $\lambda_{3}$ should not be large in the training time.

Specially, DOTA is a typical multi-scale VHR aerial dataset. To fully exploit multi-scale information of various objects, we adopt the multi-scale strategy in training and inference time for DOTA. More concretely, the original images are resized two scales {1.0, 0.5} before dividing the image into patches, and then each scale is selected from {600, 700, 800, 900, 1000, 1100, 1200} for each sample randomly as the size of a patch image’s short side. And the long side is adjusted with the original ratio in training and testing time. Similarly, for HRSC2016, we adopted the scale with {600,700,800,900} for each sample randomly as the size of a patch image’s short side. The long side is also adjusted with the original ratio in training and testing time. In addition, we adopted random flipping and random rotating at an angle from [$-90^{\circ }$,$90^{\circ }$] with $15^{\circ }$ interval for data augmentation on two datasets.

### 3.3. Evaluation Metrics

To evaluate the performance of object detector, widely used mean Average Precision (mAP) is adopted as evaluation criteria, which is calculated by Recall and Precision. The Precision metric represents the ratio of detection that are true positives, and the Recall metric means the ratio of positives that are detected accurately. The Precision and Recall metrics can be formulated as follows:

(9) $$Precision=\frac{TP}{\left(TP+FP\right)}$$

(10) $$Recall=\frac{TP}{\left(TP+FN\right)}$$

where, TP, FP and FN indicate the number of true positive, false positive and false negative respectively. Meanwhile, we can also utilize Recall (R) and Precision (P) to get Average Precision (AP) value of each category. The mAP is calculated by the mean value of AP over all categories. They are defined as follows:

(11) $$AP=\int_{0}^{1}P(R)dR$$

(12) $$mAP=\frac{1}{N_{cls}}\sum_{i=1}^{N_{cls}}AP_{i}$$

where P(R) indicates the P-R function, $N_{cls}$ represents the number of categories.

### 3.4. Comparison with the-State-of-Art Methods

In this subsection, we compare the performance of our proposed method MFIAR-Net with the state-of-art rotation detection methods on DOTA [35] and HRSC2016 [37] datasets. The generality and effectiveness of the proposed framework are demonstrated by showing exhibiting quantitative and qualitative results.

#### 3.4.1. Results on DOTA Dataset

**Figure 8:**
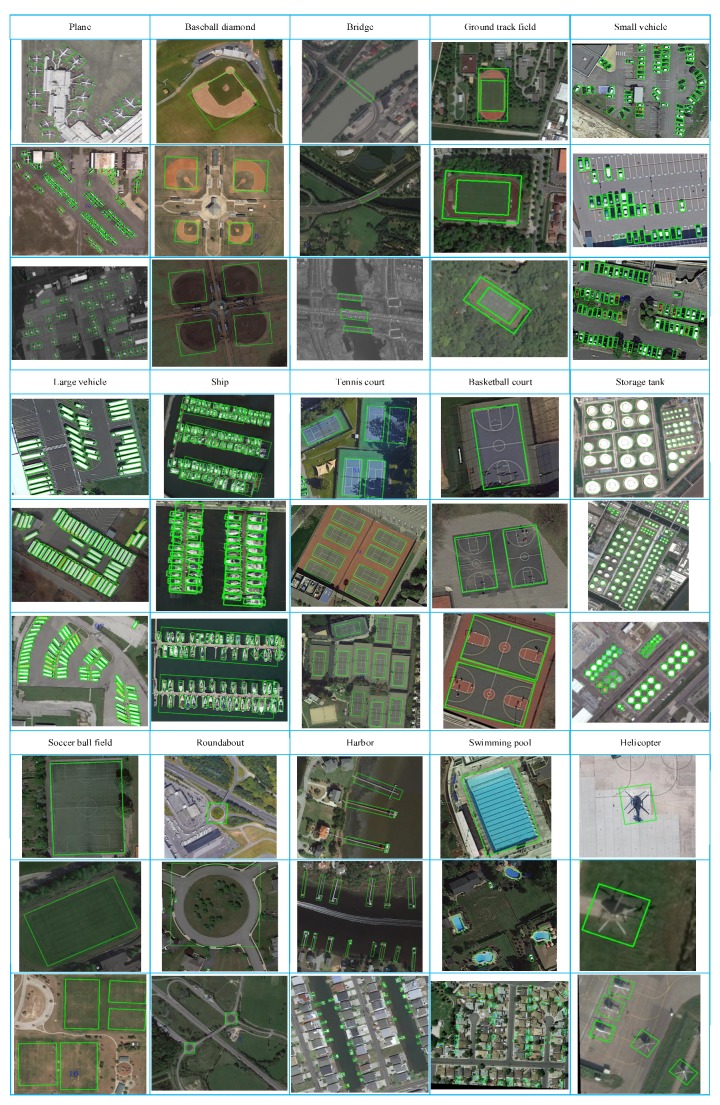
Visualization of detection results of proposed MFIAR-Net on OBB task of DOTA.

Table 1 summarizes the experimental results of different methods. As can be seen in Table 1, we compare with FR-O [35], R-DFPN [29], R${}^{2}$CNN [32], RRPN [44], ICN [30], RoI-Transformer [31] and recently proposed SCRDet [33] and RADet [34]. The results reported here were obtained by submitting our predictions to the official DOTA evaluation server. At first, the methods based on generating rotated region proposals including R-DFPN, RRPN, ICN and RoI-Transformer, can achieve a good performance with the fitting proposals produced from RPN stage. Among these methods, ICN and RoI-Transformer focus on the OBB Task of multi-class object detection for remote sensing images, which gain performance at 68.20% and 69.56% mAP. Especially for ship (SH) and ground track field (GTF), RoI-Transformer have the best AP values with 83.59% and 75.92%, respectively. R-DFPN is designed for ship detection, which have inadequate ability to detect multi-class and multi-scale objects. RRPN is constructed for rotated text scene detection, which are not suitable for aerial images with complex background. Secondly, the methods depending on regression from coarse horizontal region proposals including R${}^{2}$CNN, SCRDet and our proposed MFIAR-Net, these methods have the clear superiority in mAP. SCRDet gets 72.61% mAP and our proposed MFIAR-Net gains 73.49% mAP. These methods concentrate on the OBB regression with multi-category rotation detector. The SCRDet has best performance in plane (PL), storage tank (ST), soccer ball field (SBF) and roundabout (RA). MFIAR-Net has distinct performance in baseball diamond (BD) and small vehicle (SV) and harbor (HA). Thirdly, RADet based on rotated rectangle from the mask shape prediction, is a simple and efficient method for obtaining a rotating bounding box, which gains competent mAP performance with 69.09%. For large-vehicle (LV) and swimming pool (SP), RADet has the best AP value, which illustrates the manner calculated from mask is efficient. Compared with other methods, our proposed MFIAR-Net outperforms other advanced rotation methods, which is more accurate for multi-class object detection and more suitable for the rotated object detection in VHR aerial images. Some qualitative results of MFIAR-Net on DOTA are given in Figure 8.

#### 3.4.2. Results on HRSC2016 Dataset

**Figure 9:**
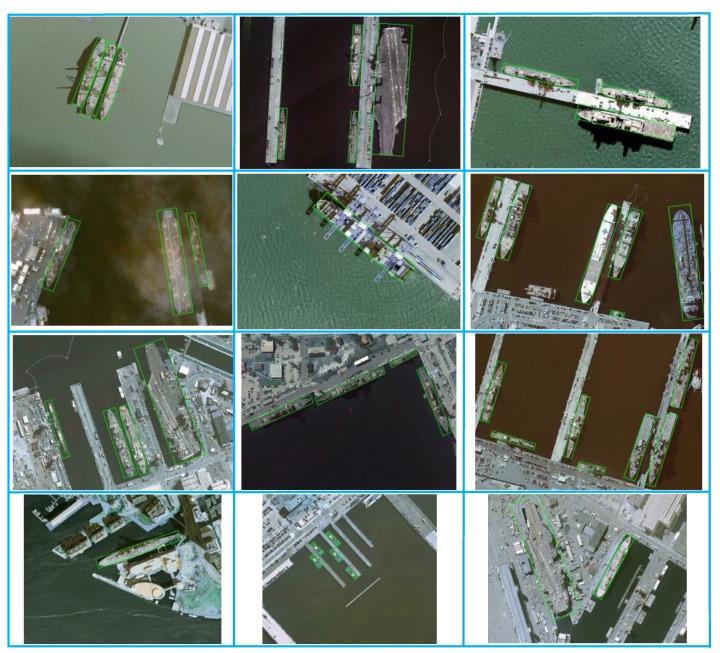
Visualization of detection results of proposed MFIAR-Net on test set of HRSC2016.

To further validate the effectiveness of our proposed rotation framework, we construct extensive experiments on HRSC2016 dataset. HRSC2016 contains a large number of thin and long ship instances with arbitrary orientation, which brings in ultimate challenge for rotation-based methods. For HRSC2016 evaluation, the results are reported by the standard VOC AP metrics with Intersection Over Union (IoU) threshold of 0.5. RoI-Transformer [31], SCRDet [33] and RADet [34] are selected as the representation in three categories of oriented object detections to make a comparison with our proposed MFIAR-Net. As the Table 2 shows, these methods achieve competitive results. Specially, RoI-Transformer [31] can achieve 86.20% mAP owing to the high-quality rotated region proposals. Compared with RoI-Transformer, our proposed MFIAR-Net increased 3.61% highly. There are two main reasons. On one hand, it’s hard to distinguish between background and foreground in most situations of HRSC2016. Our designed attention network DPFAN supervised by the binary mask plays an important role in achieving state-of-art performance. On the other hand, PS RoI Align can make the network avoid the misalignment between objects and extracted feature maps, especially for strip-like ship in HRSC2016. Some visualization results are shown in Figure 9.

In order to illustrate the computational complexity of the presented method, we make a comparison about the detection speed with different models. To make the comparison as fair as possible, we set the same image size with 800×800 and test the single image with post-processing operations (like R-NMS). As Table 2 shows, the speed of SCRDet [33] is 0.20 s, whose time consuming of computation is due to the channel of base feature map is 1024 until finish detection. The channel of our proposed MFIAR-Net is 256 after FPN, which can save much time without performance loss. RADet [34] is based on Mask RCNN [16], which prediction of mask branch is a pixel-to-pixel task. It needs more computation time with 0.24 s speed. RoI-Transformer [31] is based on generating rotated proposals in RPN stage. Compared the horizontal proposals, each pixel need to generate dozens or even hundreds of rotated proposals, which brings in more computation. In our proposed MFIAR-Net, the OBB prediction is regressed from the horizontal proposals from the RPN stage. In order to cover all objects’ size as far as possible, the setting of anchor ratios is [1/1, 1/2, 1/3, 1/4, 1/5, 1/6, 1/7, 1/9], which will result in more computation. Nevertheless, MFIAR-Net can achieve 0.14s speed, which also illustrates the proposed method has potential application.

## 4. Discussion

### 4.1. Ablation Study

In this subsection, we conduct an ablation study to analyze and discuss the impact of each component of the proposed MFIAR-Net on performance. Compared with HRSC2016, DOTA has large scale variations of multi-category objects and more complicated backgrounds of geospatial scenarios to better verify the effectiveness. For ablation experiments, we firstly to setup the baseline and then gradually to set different network configurations to make an efficient and robust framework on DOTA. Note that all experiments are trained on train and validation datasets and our predicted results of test dataset are submitted to official DOTA evaluation server to get final experimental results.

#### 4.1.1. Ablation for Multi-Scale Feature Integration Network (MFIN)

**Figure 10:**
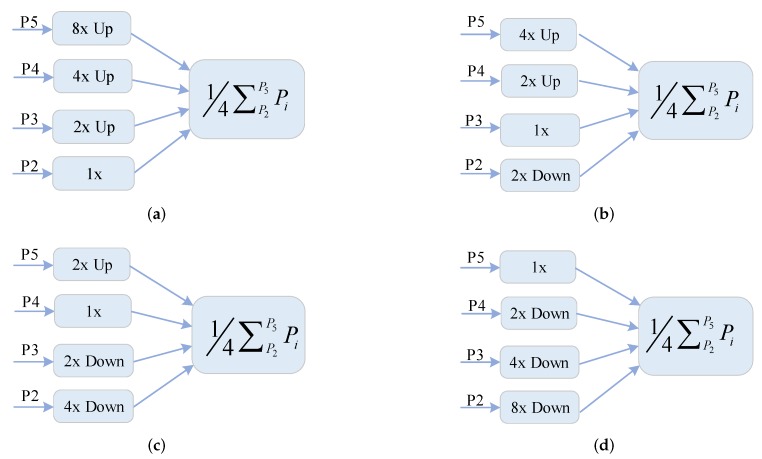
Different integration manners of multi-scale features of P2, P3, P4, P5. (**a**) The way of Integration into P2; (**b**) The way of Integration into P3; (**c**) The way of Integration into P4; (**d**) The way of Integration into P5. Note that Up and Down represent upsample and downsample operations respectively are achieved by the bilinear interpolation method.

First of all, we set the baseline to capture the multi-scale features. As we all know, FPN [14] based on Faster RCNN [6] has the strong ability to handle the multi-scale objects. R-DPFN [29] have analyzed the FPN is better than Faster RCNN with single high-level feature map. And the later related works including ICN [30], RoI-Transformer [31] and recently proposed method RANet [34] are based on FPN in feature extraction, which illustrates the advantages of multi-scale feature pyramid. So, we setup FPN model based on Faster RCNN with rotated detection head as our baseline. Then, we construct the network to improve the expressiveness of multi-scale features based on the baseline model. Inspired by the Libra RCNN [17], we find that the integration of feature pyramid representations determines the detection performance. As Figure 10 shows, we construct the network in different integration manners of multi-scale features to explore this idea. As the Table 3 shows, the performance which way of integration into P3 feature map performs the best mAP of 65.81%, 2.09% larger than baseline model. On one hand, the size of feature map is larger, the object samples are more high-quality. On the other hand, the low-level feature is more content detailed, which can bring large number of negative samples and weaken the detection performance. The main reasons why the way of integration into P3 has the best performance can divide into two aspects. Firstly, P3 can balance semantic and location feature map by integrating the pyramidal representations in the intermediate position. Secondly, the size of P3 can make the network have finer object sampling, in which negative samples is not excessive.

In addition, in order to extract more powerful features for rotated objects, the AC Blocks are adopted after each pyramidal feature instead of the standard square-kernel convolutional layer. The horizontal and vertical kernels were added to enrich the feature space, especially for the model’s adaptability of rotation distortions, which can improve the capability of rotational invariance for the network. The Table 3 shows that AC Block can reach 66.75%.

#### 4.1.2. Ablation for PS RoI Align Layer

In order to demonstrate the efforts of PS RoI Align layer, we make an ablation study for RoI Pooling, RoI Align and PS RoI Align separately. Our baseline is based on RoI Pooling layer, which divides the region proposal into a fixed-length in the second stage. It can be evidenced in Table 4 that the detection results have been improved by 1.27% and 0.72% after replacing RoI Pooling and RoI Align with PS RoI Align, respectively. The PS RoI Align layer has better performance, reaching 68.02%. On one hand, PS RoI Align can address the problem of location misalignment, compared with RoI Pooling. On the other hand, PS RoI Align can promote the sensitivity of location by means of encoding score maps with reference to a relative spatial position. By combining RoI Align and PS RoI Pooling, PS RoI Align brings their advantages to work well and make a better performance of mAP.

#### 4.1.3. Ablation for Double-Path Feature Attention Network (DPFAN)

**Figure 11:**
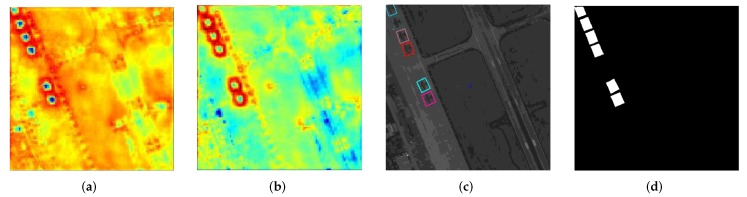
Visualization of the attention network of DPFAN. (**a**) Input feature map of DPFAN; (**b**) Output feature map of DPFAN; (**c**) Ground truth; (**d**) Binary mask map as supervised information for attention network.

Attention structure plays a key role in guiding the network to focus on the object information and suppress the influence of background. In Table 5, we study on the techniques of attention structure. The detection performance of mAP was increased by 1.55% to 69.57% after adding the proposed Double-Path Feature Attention Network (DPFAN), which demonstrates the effectiveness of including attention structure. In addition, we also construct experiments with Single-Path Feature Attention Network (SPFAN), which is constructed with the one branch of DPFAN. The Table 5 shows that SPFAN can achieve a small improvement by 0.61%. The two-branch DPFAN can obtain more supervised information to guide the network to present the object’s features better. As Figure 11 illustrates, the background is distinctly suppressed after our proposed feature attention network. It also demonstrated intuitively the effectiveness of DPFAN.

#### 4.1.4. Ablation for Multi-scale Strategy

We perform the multi-scale training and inference strategy to enhance the scale diversity of remote sensing objects. Table 6 shows that the input image with single scale of 800 can get the 69.57% mAP. At first, the original images are resized two scales {1.0, 0.5} before dividing the image into patches. After partition of the scaled images, scaling the short side of cutout image to one size from {600, 700, 800, 900, 1000, 1100, 1200} randomly and the long side at the original ratio can improve the performance of model from 69.57% to 72.51% with backbone of ResNet-101, which demonstrates the effectiveness of the multi-scale strategy in training and inference time. Furthermore, our model with a deeper backbone ResNet-152 can achieve a better 73.49% performance.

### 4.2. Discuss on HBB Task

Our proposed MFIAR-Net is designed with rotated detection head (x,y,w,h,θ) and achieves the state-of-art detection performance on OBB task. To better verify comprehensive performance of our proposed network structure, we conducted the experiments with traditional HBB detection head (x,y,w,h). It can be seen in Table 7 that MFIAR-Net also have competitive performance with HBB task on DOTA dataset, compared with the advanced methods. The experimental results show that the proposed network structure has strong generalization.

### 4.3. Limitations of Proposed Method

Although our designed method has achieved great performance for multi-scale rotation detection, there still exist some limitations. On one hand, the long and thin OBBs with acute angle come with the difficultly for optimization of the whole network. A more appropriate objective loss function needs to be explored further. On the other hand, for the particularly small objects such as the ship, our method still needs to be improved compared with RoI-Transformer. In order to boost the performance further, it can be from two aspects: one is a better network to reconcile the small and large objects, another one is a preferable OBB’s representation form for oriented objects. At the same time, detection efficiency is also an import factor in practical remote sensing system. In the future, we will concentrate more attention on the real-time detection with high-accuracy.

## 5. Conclusions

In this paper, we have proposed a novel and effective region-based rotation object detection framework named MFIAR-Net, designed expertly for multi-category and arbitrary-orientation objects in VHR aerial images. The MFIN can extract multi-scale features discriminatively and integrate into a distinguished feature with an appropriate size, which balanced semantically strong, coarse-resolution features and semantically weak, high-resolution features simultaneously. Taking the complex and diverse background into consideration, a supervised Double-Path Feature Attention Network (DPFAN) is designed to guide the whole network to capture the object information and suppress irrelevant noise. Moreover, a robust Rotation Detection Network is presented, which effectively achieves OBB’s localization and classification. The ablation study was constructed carefully to demonstrate the performance improvement of each component in the overall network. Experimental results on public datasets DOTA and HRSC2016 show that our framework can achieve state-of-art performance with a competitive detection speed on OBB task.

## Figures and Tables

**Table 1:** Comparison of the performance on OBB task with the state-of-art methods on DOTA test set. The numbers in boldface indicate the best detection results on each class.

Method	PL	BD	BR	GTF	SV	LV	SH	TC	BC	ST	SBF	RA	HA	SP	HC	mAP(%)
FR-O	79.09	69.12	17.17	63.49	34.20	37.16	36.20	89.19	69.60	58.96	49.40	52.52	46.69	44.80	46.30	52.93
R-DFPN	80.92	65.82	33.77	58.94	55.77	50.94	54.78	90.33	66.34	68.66	48.73	51.76	55.10	51.32	35.88	57.94
R${}^{2}$CNN	80.94	65.67	35.34	67.44	59.92	50.91	55.81	90.67	66.92	72.39	55.06	52.23	55.14	53.35	48.22	60.67
RRPN	88.52	71.20	31.66	59.30	51.85	56.19	57.25	90.81	72.84	67.38	56.69	52.84	53.08	51.94	53.58	61.01
ICN	81.40	74.30	47.70	70.30	64.90	67.80	70.00	90.80	79.10	78.20	53.60	62.90	67.00	64.20	50.20	68.20
RADet	79.45	76.99	48.05	65.83	65.46	**74.40**	68.86	89.70	78.14	74.97	49.92	64.63	66.14	**71.58**	62.16	69.09
RoI-Transformer	88.64	78.52	43.44	**75.92**	68.81	73.68	**83.59**	90.74	77.27	81.46	58.39	53.54	62.83	58.93	47.67	69.56
SCRDet	**89.98**	80.65	52.09	68.36	68.36	60.32	72.41	**90.85**	**87.94**	**86.86**	**65.02**	**66.68**	66.25	68.24	**65.21**	72.61
MFIAR-Net(ours)	89.62	**84.03**	**52.41**	70.30	**70.13**	67.64	77.81	**90.85**	85.40	86.22	63.21	64.14	**68.31**	70.21	62.11	**73.49**

**Table 2:** Comparison of detection accuracy and speed with advanced methods on HRSC2016 test set. The numbers in boldface indicate the best detection result.

Method	R${}^{2}$CNN	RRPN	SCRDet	RADet	RoI-Transformer	MFIAR-Net(ours)
mAP(%)	73.07	79.08	83.41	84.31	86.20	**89.81**
speed	0.5s	0.28s	0.20s	0.24s	0.16s	**0.14s**

**Table 3:** Different integration manners of the multi-scale feature maps for Multi-Scale Feature Integration Network (MFIN).

Method	mAP(%)
FPN (based on Faster R-CNN)	63.72
+Integration into P2	64.53
+Integration into P3	**65.81**
+Integration into P4	64.25
+Integration into P5	60.73
+MFIN (AC Block + Integration into P3)	**66.75**

**Table 4:** Different RoI Pooling layer. The numbers in boldface indicate the best performance.

Method	mAP(%)
Baseline (RoI Pooling) + MFIN	66.75
RoI Align	67.30
PS RoI Align	**68.02**

**Table 5:** Different feature attention network configurations. DPFAN represents our proposed Double-Path Feature Attention Network. SPFAN represents a single branch of DPFAN. The numbers in boldface indicate the best performance.

Method	mAP(%)
Baseline + MFIN + PS RoI Align	68.02
+ SPFAN	68.63
+ DPFAN	**69.57**

**Table 6:** The ablation study for the multi-scale strategy. The numbers in boldface indicate the best performance.

Method	Backbone	mAP(%)
Baseline + MFIN + PS RoI Align + DPFAN	ResNet-101	69.57
+Multi-scale Strategy	ResNet-101	72.51
+Multi-scale Strategy	ResNet-152	**73.49**

**Table 7:** Comparison of detection accuracy of different methods with HBB on DOTA.

Method	SSD [20]	YOLOv2 [19]	FR-H [6]	ICN [30]	IOU-Adaptive [45]	SCRDet [33]	MFIAR-Net(ours)
mAP(%)	10.94	39.20	60.46	72.50	72.72	75.35	**76.07**
